# Using ecoinformatics to evaluate the impact of crop and herbicide rotations on herbicide intensity

**DOI:** 10.1002/ps.70925

**Published:** 2026-05-23

**Authors:** Shlomi Aharon, Ran Nisim Lati, Hanan Eizenberg, Eitan Goldshtein, Yafit Cohen

**Affiliations:** ^1^ The Robert H. Smith Institute of Plant Sciences and Genetics in Agriculture The Hebrew University of Jerusalem Rehovot Israel; ^2^ Department of Phytopathology and Weed Research, Newe Ya'ar Research Center Agricultural Research Organization Ramat Yishay Israel; ^3^ Institute of Agricultural and Biosystems Engineering, Volcani Institute Agricultural Research Organization Rishon LeZion Israel

**Keywords:** big data, crop rotation, integrated weed management (IWM), mechanical weed control, row‐crop

## Abstract

**BACKGROUND:**

Crop rotation is a central component of integrated weed management (IWM) under real‐world conditions, yet its impact on herbicide use remains unclear. To address this challenge, we developed an ecoinformatics‐driven analysis approach based on farmer‐reported data from 11 farms, spanning approximately 400 fields and 3303 crop‐year records across diverse arable zones. The workflow analysis was divided into three main stages: (i) trend‐screening, examining temporal changes in herbicide intensity across major summer crops and rotational patterns of crops and herbicides; (ii) identification of key drivers associated with variability in herbicide intensity, and (iii) mechanistic clue finding, revealing explanations for patterns observed in the first two stages.

**RESULTS:**

A significant positive association was found between herbicide intensity and years in maize (*P* < 0.001). The frequency of cotton in crop rotation was identified as the most consistent predictor of herbicide intensity in this crop (estimate = −0.25, *P* < 0.001). Maize fields in which cotton was prevalent in the rotation received approximately one fewer herbicide application than fields without cotton in the rotation. Principal component analysis (PCA) highlighted tillage as the primary driver of the differentiation between maize fields with and without a history of cotton. Temporal testing showed that the cotton effect persisted for 2 years.

**CONCLUSION:**

Cotton‐based rotations reduced herbicide inputs in maize, with cotton‐specific tillage practices providing a mechanistic explanation for the weed‐suppressive effect. These results highlight the advantage of IWM and demonstrate the utility of ecoinformatics for detecting rotation effects in large‐scale farm data. © 2026 The Author(s). *Pest Management Science* published by John Wiley & Sons Ltd on behalf of Society of Chemical Industry.

## Graphical abstract

Maize fields with cotton in rotation receive lower herbicide application than fields without cotton.

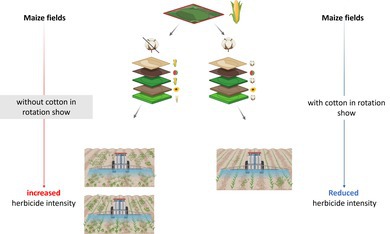

**ABBREVIATIONS**
AIC: Akaike information criterion
edf: estimated degrees of freedom
GAM: generalized additive model
GLM: generalized linear model
HCF: historical crop frequency
HHF: historical herbicide frequency
HSTF: historical soil tillage frequency
HTF: herbicide treatment frequency
MOA: mode of action
NAI: number of active ingredients
PCA: principal component analysis

## 1 INTRODUCTION

Chemical application has been the predominant weed control strategy in modern agriculture for over 60 years. However, this reliance now faces two critical challenges that undermine its long‐term viability. First, escalating chemical dependency has raised significant environmental and human health concerns. Second, the rapid, widespread evolution of herbicide‐resistant weeds, now documented in numerous crops, poses a threat to sustainable agricultural practices worldwide.^1^ Furthermore, Kniss *et al*.^2^ showed that between 1990 and 2015, herbicide use increased substantially while total arable land remained stable, suggesting that greater herbicide inputs are required to maintain the same level of weed control. This increase in chemical use, together with growing public awareness of potential human health risks and herbicide resistance, has intensified pressure to reduce herbicide dependence in agriculture. Consequently, these trends have driven a shift toward more sustainable weed management strategies, with integrated weed management (IWM) increasingly promoted as a key approach to reduce reliance on herbicides and enhance the long‐term sustainability of agricultural systems.^3^ The core of IWM lies in the systemic integration of diversified herbicide‐based programs with a set of non‐chemical weed control tactics, including mechanical, physical, and cultural^4^. Within this framework, crop rotation is a central IWM component that strengthens the sustainability weed control management. Several mechanisms are thought to contribute to the effects of crop rotation on weed control, including enhanced crop competitiveness with weeds, increased variability in tillage and crop management timing (e.g., sowing and harvest), and diversification of herbicide modes of action.^5^ However, the relative contribution of these mechanisms remains poorly resolved. In most studies, comparisons are limited to ‘simple’ *versus* ‘diverse’ rotations or to differences in rotation composition, with little elucidation of the roles of specific crops, herbicide programs, or other rotation‐associated practices.^6^, ^7^, ^8^

Another limitation of IWM and crop rotation studies relates to the spatial or temporal scale at which data were extracted. The tightly controlled conditions of research‐station trials, whether they investigate crop and herbicide rotations or other management strategies, may not necessarily be relevant to commercial fields over the long term. With the changing large‐scale commercial cropping systems, overlapping decisions regarding tillage timing, inter‐row cultivation, double cropping, and spray scheduling can confound the effects of rotations or herbicides tested in research‐station trials.^9^, ^10^, ^11^ Resolving the above uncertainties calls for an analysis that captures the full heterogeneity of real‐world farming practice rather than the simplified conditions of experimental plots.

In agriculture, ecoinformatics addresses the challenge of studying phenomena, management processes, and their associated effects across broad spatial and temporal scales under real‐world conditions by integrating increasingly available farmer‐generated data across crops and management contexts into a single comprehensive database.^12^, ^13^, ^14^, ^15^, ^16^ For example, an analysis of over 1400 field‐year records for cotton showed that the preceding crop in a crop‐rotation scheme influenced pest density.^17^ Specifically, alfalfa and sugar beet in the previous season increased *Lygus hesperus* density in the field and reduced cotton yield, whereas tomato had the opposite effect. Similarly, for weed science, ecoinformatics offers new opportunities to quantify infestation dynamics and evaluate management outcomes under commercial field conditions. Another study on the spatial spread of Egyptian broomrape (*Orobanche cumana* Wallr.) demonstrated that infestation patterns were shaped by crop rotation, infestation history, and proximity to previously infested fields.^18^ More recently, Gafni *et al*.^19^ showed that *Amaranthus* control depends on both management and environment: pre‐plant sulfosulfuron and intensive management improved control, while precipitation had the opposite effects depending on its timing relative to planting.

However, other weed use‐related ecoinformatic studies have limitations in field‐level detail resolution and do not relate to herbicide usage.^20^ Givens *et al*.,^21^ for example, rely on farmers' survey data to describe herbicide‐use patterns and perceptions at a single‐season scale, without resolving field‐level crop rotation histories, whereas Andert *et al*.^22^ used 11 years of on‐farm data to show that diversified crop sequences reduce total herbicide costs and improve economic outcomes but did not explicitly quantify herbicide use intensity. Consequently, to date, no study has combined multi‐year farm records that integrate both crop sequence and detailed herbicide application histories to assess how specific crops and herbicide rotations influence herbicide intensity in commercial farming systems. Data from commercial farms in Israel can help address this lacuna. Local summer cropping systems are unusually diverse in that >90% of commercial fields rotate ≥3 crops within 5 years (Supporting Information, Fig. S1), sometimes even under double cropping. Therefore, demonstrating a rotation effect despite this baseline diversity would provide a strong test of its agronomic relevance. In this context, this study aimed to integrate crop, herbicide and mechanical practices histories, into a single farmer‐sourced database to enable a field‐scale assessment of how crop rotation patterns and herbicide active ingredient histories are associated with herbicide use intensity under the highly diversified rotations typical of Israeli agriculture, thereby providing insights into the mechanisms driving the effectiveness of IWMs.

## 2 MATERIALS AND METHODS

This study implements a three‐stage ecoinformatics workflow to determine how crop‐rotation choices and herbicide‐mode histories in a highly diversified cropping system influence herbicide application intensity. Analysis is based on 7 years (2015–2022) of detailed management records from 3303 field‐years across 11 farms spanning different agroclimatic zones in Israel. The analysis proceeded in sequential stages;

### 2.1 Stage 1 – trend screening

We first examined whether herbicide intensity increased in Israel's main summer crops over the 7 years under study. This stage was informed by global evidence that herbicide use over the past few years has increased in herbicide‐reliant cropping systems.^1^, ^2^ Accordingly, we formulated the following hypothesis (H1): Between 2015 and 2022, herbicide intensity increased in the main summer crops in Israel.

### 2.2 Stage 2 – identification of key drivers

In this stage, we tested whether the recent five‐year histories of specific crops and herbicide applications explain field‐level herbicide intensity. This stage was informed by local knowledge, particularly insights from farmers and agronomists, suggesting that crop types differ in their effects on weed infestation. For example, cotton is viewed as a crop that promotes ‘lower weed infestation’,^23^, ^24^ whereas watermelon is known as a crop that ‘promotes weed infestation’.^25^ Therefore, we hypothesized (H2a) that cotton in a crop‐rotation history is associated with reduced herbicide intensity in the following crop, whereas watermelon is associated with increased intensity.

While crop history may influence weed pressure, past herbicide use patterns may also shape long‐term weed‐control outcomes, as some herbicide active ingredients may be associated with more effective control, whereas others may be associated with reduced efficacy and, over time, greater herbicide reliance.^26^ Therefore, we hypothesized (H2b) that the historical frequency of herbicide active ingredients is associated with differences in herbicide intensity, without considering whether the effect increases or decreases herbicide intensity.

### 2.3 Stage 3 – mechanistic clue finding

Finally, considering the results from the two preceding stages, we explored why cotton in the history of crop rotation appears to reduce herbicide use and how long the benefit lasts. Deep plowing is mandatory following cotton harvest in Israel, and it is known to bury small‐seeded weeds below emergence depth.^24^, ^27^ Thus, we posit (H3a) that cotton‐inclusive rotations differ from cotton‐free rotations mainly through deeper or more frequent soil disturbance and thereby mediating weed seedbank reduction. Since seedbank half‐life studies suggest that such effects decline over time,^28^, ^29^ we hypothesized (H3b) that the herbicide‐intensity‐reducing influence of a cotton phase diminishes with time.

**Figure 1:**
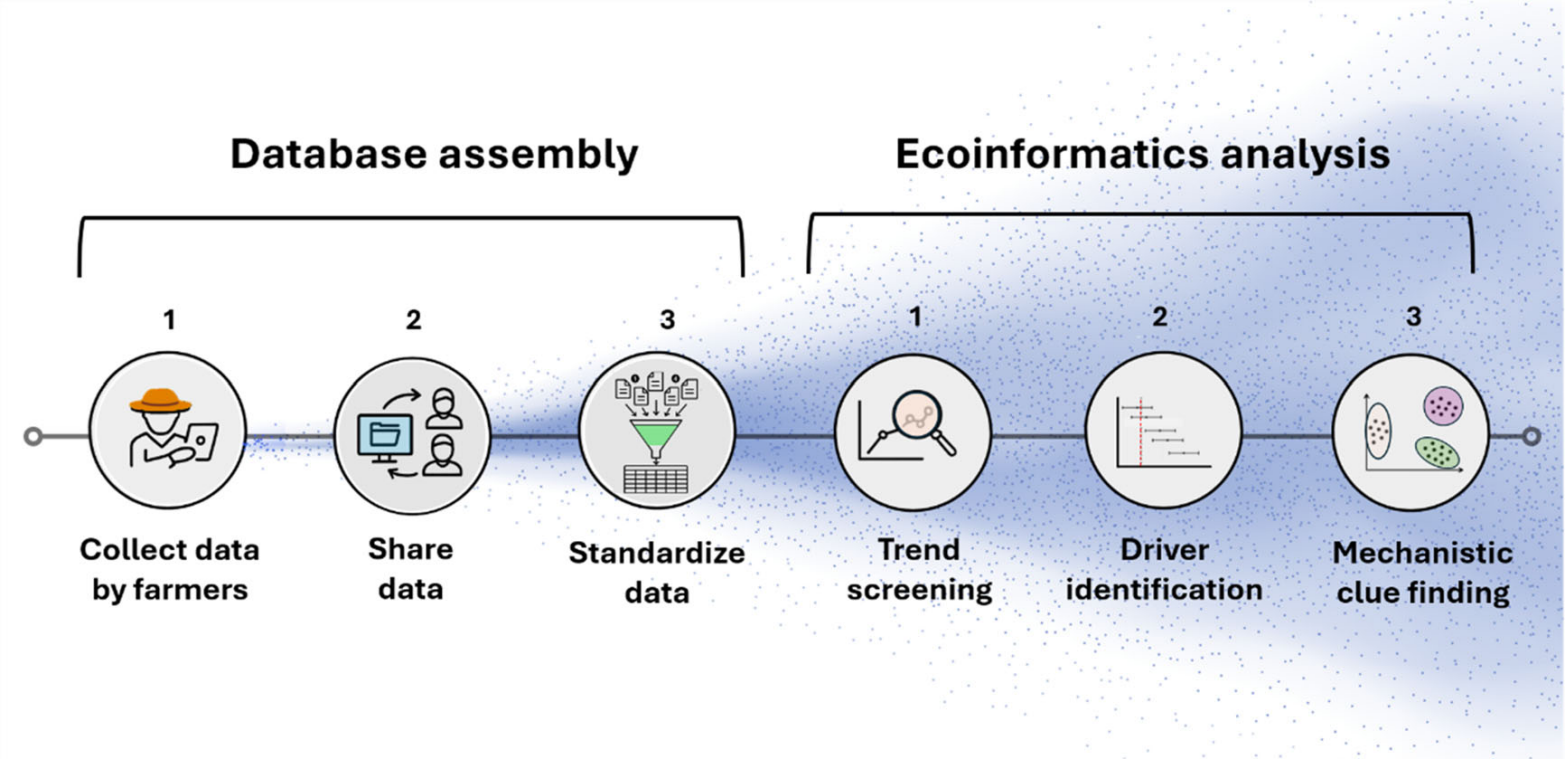
Study workflow illustrating the database assembly phase and the subsequent ecoinformatics stages of data analysis.

The three‐stage ecoinformatics workflow described above was preceded by a database assembly stage that involved extensive data collection based on farmer‐reported field operations and record keeping. The data collected from the farmers was subsequently shared with research teams and standardized to ensure consistency across farms and years. Together, the database assembly and the ecoinformatics analysis constitute the complete workflow, as illustrated in Fig. 1.

### 2.4 Data collection

**Figure 2:**
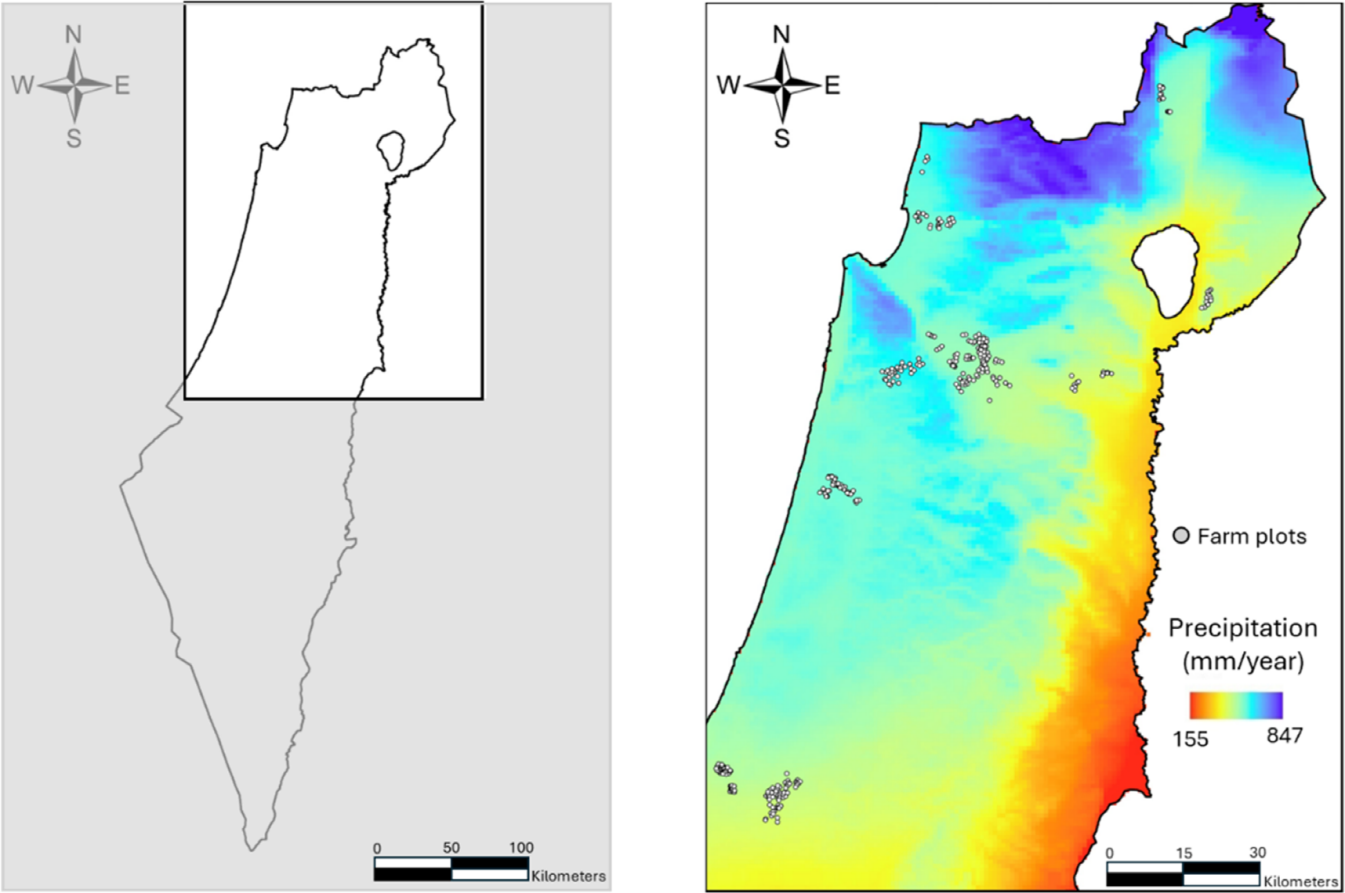
Map of Israel showing the approximately 400 fields distributed across the 11 farms used for this study. The color gradient illustrates annual precipitation levels (mm/year), based on CHELSA data for 2018.

The database was constructed by collecting historical records from 11 commercial farms located across the major agricultural regions of Israel (Fig. 2). Data were obtained directly from farmers' computers, with some farms maintaining records directly in Excel files and others exporting them from farm management applications to Excel files. The data were originally recorded independently by farmers for their own operational monitoring, without any prior coordination or standardization for research purposes. These records did not include variables such as soil type, irrigation methods, or climate conditions, thereby limiting our ability to control for environmental variations. We therefore focused on summer crops, because fewer factors interfere with herbicide application, as opposed to winter crops, for which environmental conditions can prevent access to fields and hinder necessary herbicide applications. Summer conditions thus typically facilitate better grower responsiveness and timely herbicide application. Our database covered three major summer crops in Israel: maize (commercial and sweet; encompassing 20% of the summer crops' area in Israel), watermelon (grown for confectionary seed production for human consumption, accounting for approximately 10% of the summer crop area in Israel), and cotton (encompassing 20% of the summer crops' area in Israel), which is cultivated on most fields. To assess the generalizability of our findings, we analyzed interannual rainfall (2015–2022) from three representative regions, selected on the basis of the geographic distribution of the farm fields in the database. The variability in rainfall across the years suggests that the data reflects broader climatic trends rather than specific local weather events, supporting the generalizability of our findings across different regions (Supporting Information, Table S1).

#### 2.4.1 Database assembly

A standardized database was developed to enable integration of the different sources. The resulting database contained records from 425 unique fields comprising 3303 field‐year records, over the years spanning 2015 to 2022. All recorded parameters were organized into two main categories: Raw variables, directly derived from farmers' records, and calculated indexes, developed from those records to capture management intensity, diversity, and historical patterns over time. Four raw variables and five calculated indexes were used in our three‐stage analysis, as follows:

##### 2.4.1.1 Raw variables

1. *Crop details*: This variable comprised crop name, variety, and production purpose.
2. *Field ID*: Each field was assigned to a unique ID so that we could identify repeated observations for the same field in different years.
3. *Farm name*: Fields were clustered into farms, as named by the farmers who owned the fields.
4. *Year*: The year in which the crop was grown was recorded for each field‐year.

##### 2.4.1.2 Calculated indexes

###### 2.4.1.2.1 Historical soil tillage frequency (HSTF)

Tillage data was available for approximately 60% of the fields, including detailed records of operation dates and types (cultivator, inter‐row cultivator, moldboard plow, and subsoiler). To capture long‐term tillage patterns, a separate 5‐year frequency index was calculated for each tillage operation type in fields with available records. These operation‐specific indexes were used as explanatory variables in GLM to test hypothesis H3a, which posits that more frequent tillage or greater depth of tillage is associated with reduced weed seedbank persistence and, consequently, lower herbicide intensity over time. The HSTF index was calculated as follows:

(1) $$\text{HSTF}=\sum_{i=1}^{n}\text{applied soil tillage days}$$

where n represents the number of distinct days on which a specific tillage operation (cultivator, inter‐row cultivator, moldboard plow, or subsoiler) was recorded within the five‐year window. The index was computed only for fields from 2020–2022, which ensured capture of a full five‐year history (from 2015 onward). For example, a field observed in 2020 incorporated records from 2015–2019, whereas a field observed in 2019 would have had only 4 years of history available (2015–2018), and was therefore excluded.

###### 2.4.1.2.2 Historical crop frequency (HCF)

For each field, historical crop data was available for up to seven previous years. The major crops included wheat (*Triticum aestivum* L.) (33%), cotton (*Gossypium hirsutum* L.) (16%), maize (*Zea mays* L.) (15%), watermelon (*Citrullus lanatus* L.) (8%), chickpea (*Cicer arietinum* L.) (5%), sunflower (*Helianthus annuus* L.) (3%), clover (*Trifolium subterraneum* L.) (3%), oat (*Avena fatua* L.) (3%), and pea (*Pisum sativum* L.) (3%), accounting for over 90% of the database (Supporting Information, Fig. S2). HCF was calculated for each 5‐year window as the number of times each main crop was grown. This variable was used as an explanatory variable to test hypothesis H2a, which posits that different crops in the rotation are associated with a change in herbicide intensity in the subsequent season, reflecting their differing effects on post‐harvest weed infestation levels. The HCF index was calculated as follows:

(2) $$\mathrm{HCF}=\sum_{i=1}^{n}\text{crop growing seasons}$$

where n is the number of seasons a specific crop was recorded within the preceding five‐year window for each field. The index was computed only for fields from 2020–2022, allowing capture of a full 5‐year history (from 2015 onward).

###### 2.4.1.2.3 Historical herbicide frequency (HHF)

To capture long‐term herbicide use patterns, a 5‐year frequency index was calculated separately for each herbicide active ingredient. Glyphosate was excluded from this calculation, as it is used primarily for off‐season field preparation rather than in‐season weed control. The HHF reflects the number of times a specific active ingredient was applied within the preceding 5‐year window for each field. The herbicide active ingredients showing the most pronounced temporal shifts were selected as explanatory variables to test hypothesis H2b, which posits that the variation in the historical frequency of specific herbicide active ingredients is associated with differences in subsequent herbicide intensity, reflecting differences in their long‐term effectiveness and persistence in controlling weed infestations. The HHF index was calculated as follows:

(3) $$\mathrm{HHF}=\sum_{i=1}^{n}\text{specific active ingredients}$$

where n is the number of applications in which a specific herbicide active ingredient was recorded within the preceding 5‐year window for each field. The index was calculated only for fields from 2020–2022, allowing the capture of a complete 5‐year history (from 2015 onward).

###### 2.4.1.2.4 Number of active ingredients (NAI)

This index was used to represent the diversity of herbicide active ingredients used over time.^30^ For each field, the NAI was computed within 5‐year windows as the total number of distinct herbicide active ingredients recorded, where each active ingredient was counted only once, regardless of how many times it was applied or whether it appeared in different products or tank mixes. In contrast to the HHF, which quantifies the number of applications of a specific active ingredient, the NAI reflects the diversity of herbicide MOAs used in each period. The NAI was used as an explanatory variable to test hypothesis H2b, which proposes that a greater diversity of herbicide active ingredients (higher NAI values) reflects more sustainable chemical strategies and is therefore associated with lower herbicide intensity. The NAI was calculated as:

(4) $$\mathrm{NAI}=\sum_{i=1}^{n}\text{active ingredients}$$

where n is the number of distinct herbicide active ingredients recorded within the preceding 5‐year window for each field. The index was calculated only for fields from 2020–2022, allowing the capture of a complete 5‐year history (from 2015 onward).

###### 2.4.1.2.5 The response variable, herbicide treatment frequency (HTF)

Herbicide intensity was estimated in terms of the HTF index, which reflects the total number of distinct days on which herbicide applications were implemented within a single growing season.^30^ This variable includes both pre‐emergence and post‐emergence treatments, regardless of the number of active ingredients or formulations applied on a given day. For example, if a farmer applied a mix of three herbicides on 1 day, it was counted as a single treatment day. This method was applied uniformly across all farms and fields. The HTF was calculated as follows:

(5) $$\mathrm{HTF}=\sum_{i=1}^{n}\text{applied herbicide days}$$

where n is the number of distinct days on which herbicide applications were recorded for a given crop in a given season for each field. HTF served as the response variable in the statistical models and was used as a proxy for the intensity of herbicide use, thereby providing insight into the extent to which farmers rely on herbicide‐based weed control.

### 2.5 Data analysis: the three‐stage ecoinformatics workflow

#### 2.5.1 Stage 1 – trend screening

Trend screening was divided into three main parts: (i) identifying patterns in herbicide intensity across major summer crops, (ii) analyzing changes in historical crop composition within rotations, and (iii) analyzing changes in historical herbicide use within rotations.

##### 2.5.1.1 Identifying temporal patterns in HTF

To test the first hypothesis (H1) that herbicide intensity increased in maize, watermelon and cotton fields between 2015 and 2022, we evaluated trends in HTF over this 7‐year period. Temporal trends in HTF were modeled using generalized additive models (GAM) implemented in the *mgcv* package in R.^31^ GAMs were chosen because they offer flexibility in modeling non‐linear relationships between a response variable and predictors, making them suitable for modeling ecological and agronomic data where linear assumptions may not hold.^32^ Given that year‐to‐year changes are often irregular in ecological and agronomic data, GAMs are well‐suited to identify underlying trends. GAM models use a quasi‐Poisson error distribution, suitable for the count‐based HTF and justified by model‐diagnostic tests indicating under‐dispersion, making the standard Poisson model inappropriate.^33^ The ‘Year’ variable as defines is a smooth term. Incorporating ‘Farm Name’ as a fixed effect in the model allows the evaluation of the observed trends over the years' within‐farm variations and reduces the differences that might occur across farms.^34^ For each crop, individual models were fitted,^31^ and as an example of the full model structure, the model examining the influence of ‘Year’ on HTF in maize was specified as:

$$\mathrm{gam}\_\text{corn}<\begin{array}{l} -\mathrm{gam}(\mathrm{HTF}\sim s\left(\text{Year},k=8\right)+\text{Farm Name},\text{data}\\ =\text{maize},\;\text{family}=“\text{quasipoisson}”). \end{array}$$

This structure was applied consistently across all three crops: cotton (*n* = 503), maize (*n* = 481), and watermelon (*n* = 189). The *gam. check()* function was used to assess homoscedasticity, residual distribution, and the adequacy of the smoother basis dimension. All models had k‐index values close to 1, with a *P* <0.05, indicating potential underfitting. However, the estimated degrees of freedom (edf) were substantially below the specified basis dimension (k), and increasing k had a negligible effect on the fitted values, suggesting that the basis dimension was adequate and model assumptions were not violated.^31^ Accordingly, the temporal smooth term was evaluated using *P*‐values from the model, while F‐statistics and edf were used to assess the presence, shape, and strength of temporal trends, as reported in the summaries of the GAM. We note here that the F‐statistic compares the variance explained by the smooth term to the residual variance, indicating whether the temporal trend significantly improves the model fit. A high F‐value reflects a strong temporal effect. The edf demonstrates the complexity of the smooth term, with values below 2 typically being interpreted as indicating a stable or nearly linear trend.

##### 2.5.1.2 Analysis of changes in crop and herbicide composition within rotations

Changes in crop and herbicide composition from 2015 to 2022 were analyzed across all major crops and herbicide active ingredients by calculating the annual proportion of each within its respective category. Crop trends were evaluated based on the total area planted with each crop per year, whereas herbicide trends were assessed according to the total annual number of applications of each active ingredient across all crops. Multiyear trends were analyzed separately for crops and for herbicides, ensuring that comparisons were made within each group (i.e., crop *vs*. crop and herbicide *vs*. herbicide). For example, if Crop X increased while Crop Z decreased across certain years, and later their trends were reversed, the two display a strong negative correlation. The same logic was applied to herbicide active ingredients. The correlations were quantified using Pearson correlation coefficients, calculated across years for each pair of crop and herbicide variables. The results were visualized as correlation matrices using the *ggcorrplot* package in R.^35^

As stated, negative correlations indicate opposing trends—when one variable increases, the other decreases, highlighting trade‐offs in use/cultivation. In contrast, positive correlations suggest that two variables tend to increase or decrease together. Correlations with |r| > 0.8 were considered strong, indicating aligned or opposing trends. For these variables, we assessed directionality by calculating the slope of a linear regression curve, with ‘Year’ as the independent variable and ‘Usage proportion’ as the dependent variable.

#### 2.5.2 Stage 2 – identification of key drivers

To test the twofold second hypothesis (H2), we used a Poisson generalized linear model (GLM), with explanatory variables representing the number of seasons for each crop and the herbicide active ingredients that had been applied over the previous 5 years in each field (Table 1). This GLM allowed us to assess whether the cultivation of specific crops (cotton or watermelon) in a field's recent history affected the HTF (H2a) and whether herbicide‐use histories likewise influenced HTF (H2b). Herbicide selection was based on ‘Stage 1 – trend screening,’ in which we identified the active ingredients that were associated with the most pronounced temporal changes in usage intensity across years. The GAMs that were initially tested allowed flexible modeling of potential non‐linear effects. However, diagnostic checks using the *gam. check()* function showed that the edf for most smooth terms was close to 1 or effectively zero, and the basis dimension checks (k‐index) indicated no undersmoothing (Supporting Information, Table S2), thereby suggesting that the relationships between the predictors and the response were largely linear or negligible. Therefore, a GLM with ‘Farm Name’ included as a fixed effect was selected as a more appropriate and interpretable model. GLM assumptions were assessed using Pearson residuals and the DHARMa package.^36^ All models showed no evidence of overdispersion (overdispersion ratios <1), and residual diagnostics confirmed the suitability of the Poisson distribution, with no signs of zero inflation, non‐uniformity, or outlier bias. Models were run separately for maize, cotton, and watermelon. As an example of the full model structure, the model examining influences on HTF was:

**Table 1:** Explanatory variables included in the generalized linear models (GLM)

Variable category	Specific variable	
Historical crop frequency (HCF) – Number of growing seasons over the previous 5 years in each field	Cotton
	Maize
	Watermelon
	Chickpea
	Sunflower
	Wheat
	Pea
	Oat
Historical herbicide frequency (HHF) – Number of active ingredient applications per season over the previous five years in each field (for herbicides with a major temporal change).	Pyraflufen
	Atrazine
	Clethodim
	Trifluralin
	Forasulam
	Tembotrione

glm(*HTF* ~ *maize* + *cotton* + *chickpea* + *watermelon* + *oat* + *sunflower* + *pyraflufen* + *trifluralin* + *atrazine* + *clethodim* + *florasulam*.*halauxifen* + *tembotrione* + *Farm Name*, family = Poisson, data = maize).

Each explanatory variable reflects the frequency of cultivation of a particular crop or herbicide use over the previous 5 years. The explanatory variables support formal model selection and inference through tools such as the Akaike information criterion (AIC) and allow for direct estimation and comparison of coefficients across models.^37^ In addition, GLMs are computationally efficient and stable when dealing with many predictors, making them suitable for multi‐model inference and model averaging approaches used to assess variable importance.^38^ To address potential multicollinearity among predictors, we examined Pearson correlation coefficients by using the *MuMIn* package in R.^39^ This analysis revealed a strong correlation between cotton growing area and use of the herbicide pyrithiobac. Therefore, pyrithiobac was excluded from further analysis. Model comparison was conducted using AIC to evaluate the relative support for three competing models: herbicide frequency, crop frequency, and the two frequencies combined.^39^ The relative importance of each variable was then assessed using model averaging across all candidate models. Final coefficient estimates were calculated as weighted averages using model‐specific coefficients and AIC weights (AICw), where variables not included in each model were assigned a coefficient of zero.^38^

#### 2.5.3 Stage 3 – mechanistic clue finding

##### 2.5.3.1 Revealing the mechanism behind the effect of cotton on HTF

Based on the GLM results presented in Section 2.2.3, the only variable significantly associated with herbicide management efficacy was the number of times cotton was grown in the same field during the previous 5 years. To assess the third hypothesis (H3a)—that including cotton in crop history reduces HTF through associated soil tillage practices—we conducted a separate analysis focusing on maize. Maize fields were grouped according to whether or not a cotton crop was included in their rotation history. For each group, we calculated the 5‐year frequency of moldboard and sub‐soiler plowing, inter‐row cultivation, and double cropping. To assess the diversity of herbicide strategies, we also calculated the herbicide NAIs. To explore potential underlying patterns and co‐variation among these historical management features, we applied principal component analysis (PCA), a dimensionality reduction technique well‐suited to multivariate data. With the aid of PCA, complex management histories were summarized into a few interpretable axes of variation, facilitating comparison between cotton‐inclusive and cotton‐free fields. The analysis was conducted using the *FactoMineR* package in R.^40^

##### 2.5.3.2 Characterizing the effect of cotton on HTF over time

To test hypothesis H3b that the HTF‐reducing effect of a cotton phase diminishes over time, we analyzed how long the influence of historical cotton cultivation persisted in subsequent crops. Based on seedbank half‐life studies,^28^, ^29^ we calculated the number of years since cotton had last been grown in each field and then grouped the maize fields accordingly (1, 2, 3, or 4 years since cotton cultivation). We then compared HTF values across these groups using the Wilcoxon rank‐sum test to assess whether the reduction in herbicide intensity declined and disappeared within three seasons following a cotton phase. In addition, the impact of time was evaluated using a risk index, defined as the ratio of fields with HTF > 2 to the total number of fields within the same time interval as follows:

(6) $$\text{Risk}=\frac{\text{Plots with}\;\mathrm{HTF}>2}{\text{Total plots}}$$

## 3 RESULTS

### 3.1 Stage 1 ‐ trend screening

#### 3.1.1 HTF trends over time

**Figure 3:**
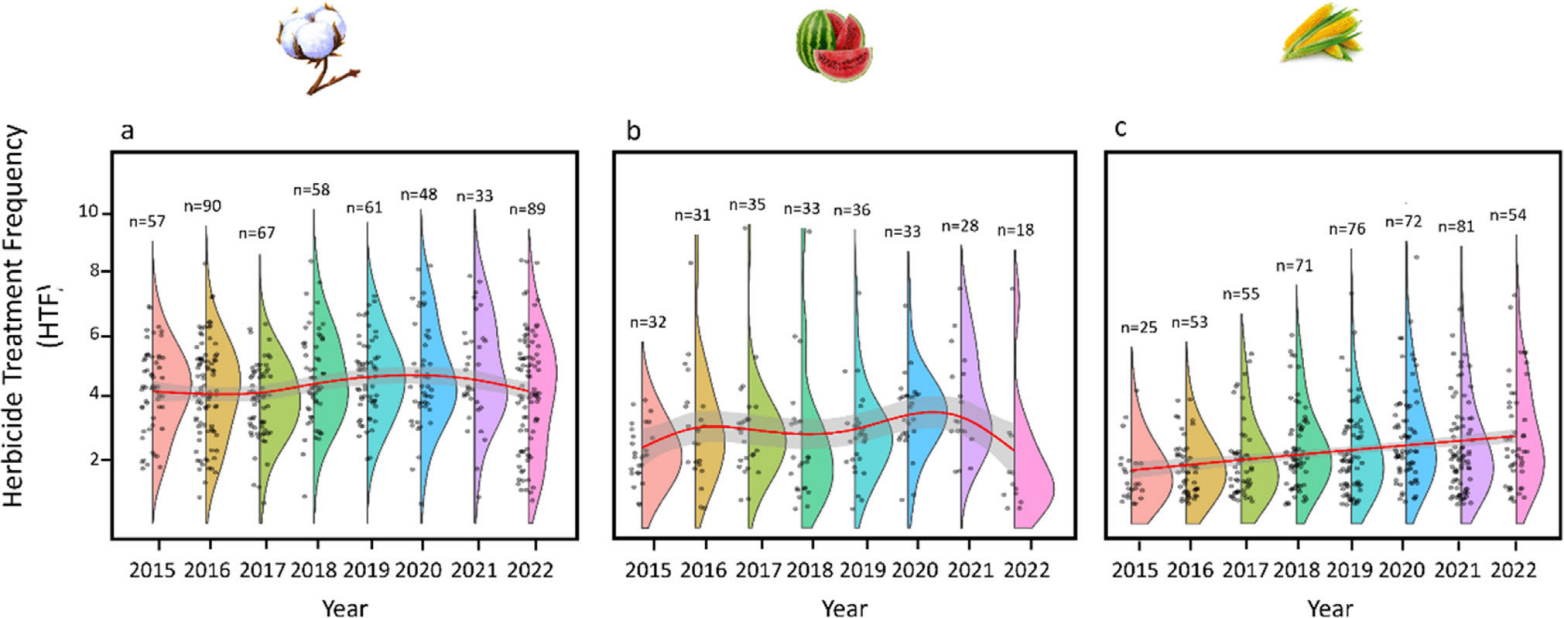
Time series of HTF in cotton (a), watermelon (b), and maize (c). HTF frequency distribution for each year is represented by distinct color clusters. Red lines represent GAM predictions; shaded regions are 95% confidence intervals; n indicates the number of crop fields sampled each year.

Over the years 2015 to 2022, HTF significantly varied over time in cotton (*P* = 0.02), watermelon (*P* < 0.001), and maize (*P* < 0.001) (Fig. 3; Supporting Information, Tables S3–S5). However, temporal patterns differed across crops. In cotton and watermelon, the GAM models indicated non‐linear and inconsistent trends (edf = 3.2, F = 2.80; edf = 4.8, F = 4.9, respectively), with modest increases in HTF between 2018 and 2020, followed by declines through to 2022 (Fig. 3(a), (b)). In contrast, maize showed a more consistent and pronounced increase in HTF, with a near‐linear pattern (edf = 2.02) and a stronger size effect (F = 12.49), indicating a sustained rise over the study period (Fig. 3(c)). This increase corresponds to a rise in herbicide use intensity from a mean HTF of 1.6 in 2015 to 2.77 in 2022 (Supporting Information, Fig. S3), indicating a substantial intensification of herbicide use in maize systems. These results are consistent with hypothesis H1 for maize, which showed a clear and sustained increase in HTF, while the inconsistent trend observed in watermelon partially contradicts the hypothesis.

#### 3.1.2 Changes in crop frequency

**Figure 4:**
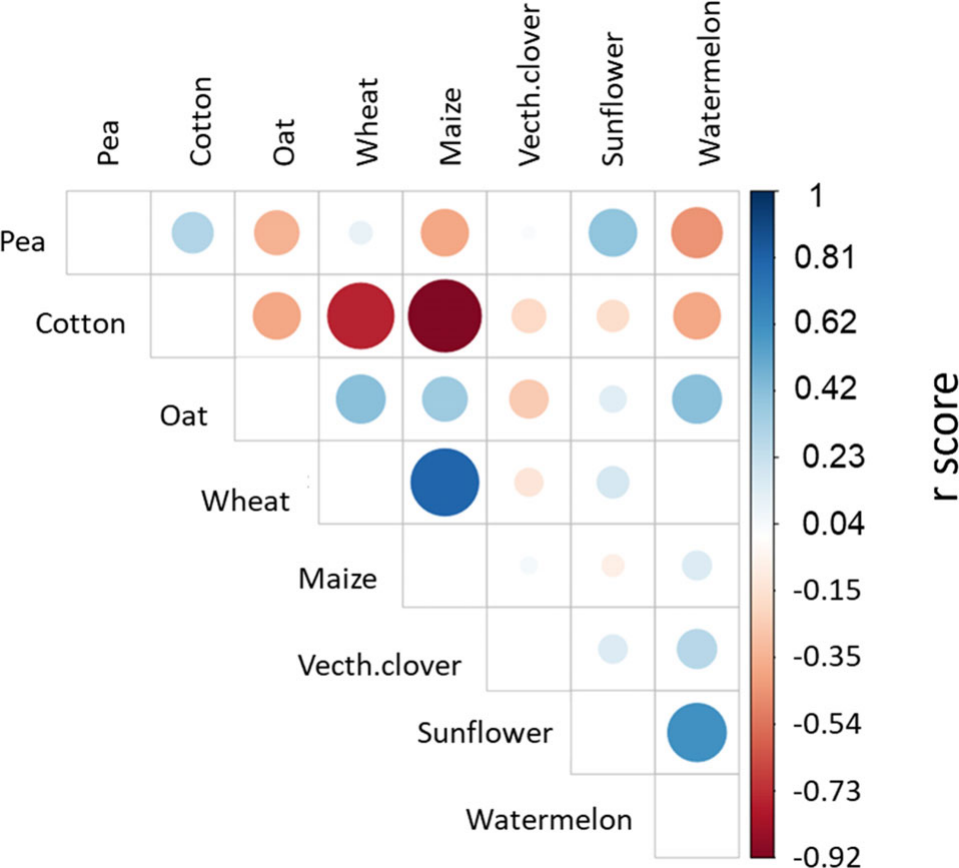
Pearson correlation matrix showing relationships between crop areas (hectarage) from 2015 to 2022. Negative correlations are shown in red, positive correlations, in blue. Circle size and color intensity represent the magnitude of the correlation coefficient (r, ranging from −1 to 1).

Figure 4 illustrates a marked shift in cultivated hectarage, with strong negative correlations between cotton and maize (r = −0.92) and cotton and wheat (r = −0.77), indicating substantial changes in crop rotation. The negative correlation between wheat and cotton is partly explained by the strong positive correlation between maize and wheat (r = 0.80), which are often grown sequentially in intensified double‐cropping systems. To quantify these trends over time, we applied linear regression models using years as the predictor and the number of fields per crop as the response variable. The results showed a 6.5% annual decline in cotton fields and a 10% annual increase in maize fields between 2015 and 2021 (Table 2). In 2022, this trend was reversed, with a sharp rise in cotton and a notable decrease in maize (Supporting Information, Fig. S4).

**Table 2:** Main temporal trends and shifts of crop area growth between 2015 and 2022. Arrows indicate the direction of change across years (↑ increase, ↓ decrease).

Trend	r score
Maize (+10%) ↑ + Cotton (−6.5%) ↓	−0.92
Wheat (+6.4%) ↑ + Cotton (−6.5%) ↓	−0.77
Wheat (+6.4%) ↑ + Maize (+10%) ↑	0.8

#### 3.1.3 Changes in herbicide frequency

**Figure 5:**
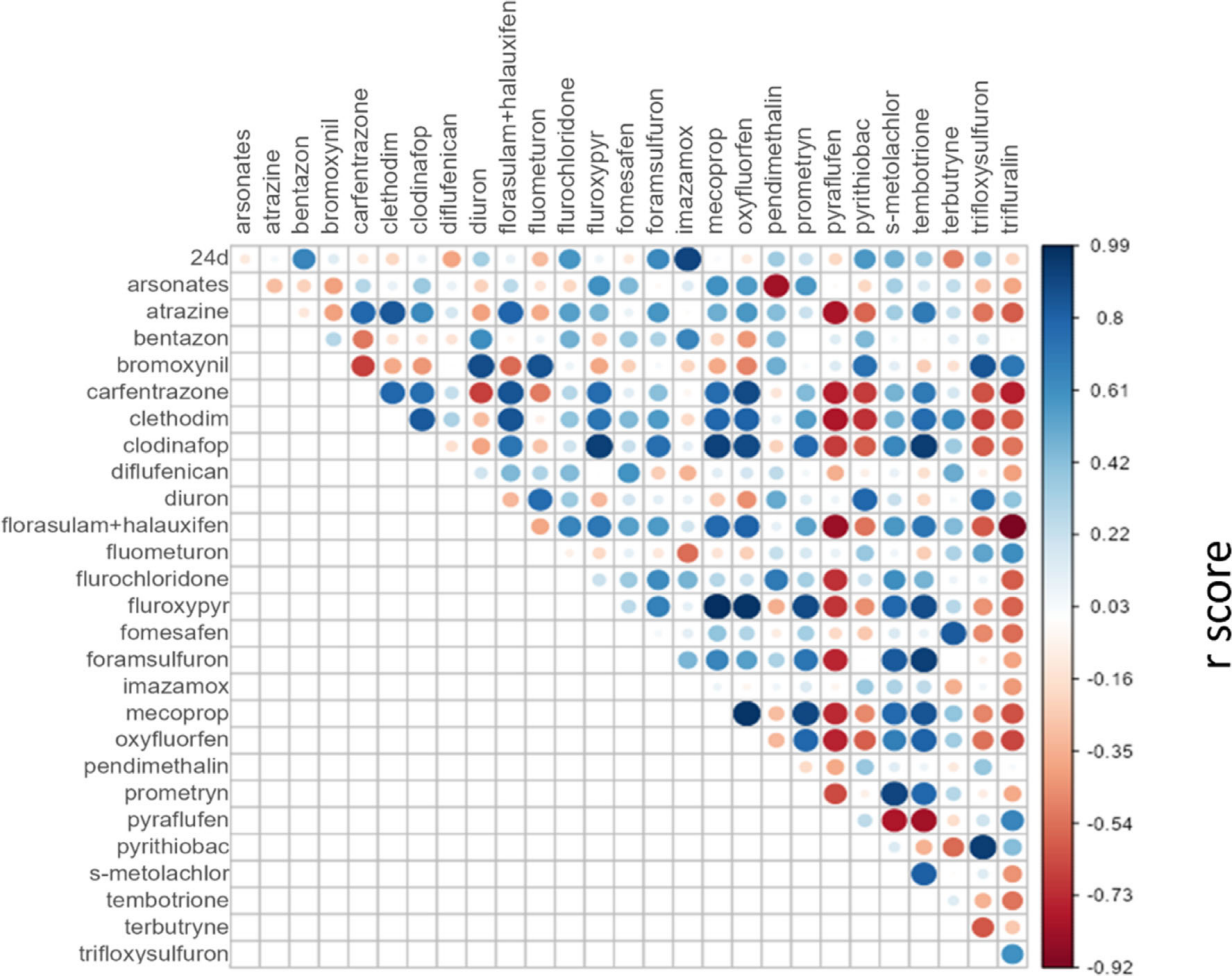
Pearson correlation matrix of applications of the main herbicide active ingredient from 2015 to 2022. Negative correlations are shown in red, positive correlations, in blue. Circle size and color intensity represent the magnitude of the correlation coefficient (r, ranging from −1 to 1).

The most notable change in herbicide use was evident in the application of pyraflufen (Fig. 5), the most widely used herbicide active ingredient in cotton fields. There were strong negative correlations between pyraflufen and five other active ingredients (r < −0.75), suggesting a shift in herbicide strategies (Table 3). There were also negative associations between trifluralin and two other ingredients, while tembotrione, oxyfluorfen, carfentrazone, and atrazine each correlated negatively with one other ingredient. Table 3 summarizes the correlation matrix and provides an overview of temporal trends for the main active ingredients.

**Table 3:** Main temporal trends and shifts in applications of herbicide active ingredient between 2015 and 2022. Arrows indicate the direction of change across years (↑ increase, ↓ decrease).

Trends	r score
Florasulam (+16.5%) **↑** + trifluralin (−14.2%) **↓**	−0.94
Florasulam (+16.5%) **↑** + pyraflufen (−7.4%) **↓**	−0.84
Tembotrione (+10.9%) **↑** + pyraflufen (−7.4%) **↓**	−0.82
Atrazine (+12.0%) **↑** + pyraflufen (−7.4%) **↓**	−0.8
Carfentrazone (+13%) **↑** + pyraflufen (−7.4%) **↓**	−0.76

### 3.2 Stage 2 – identification of key drivers

**Figure 6:**
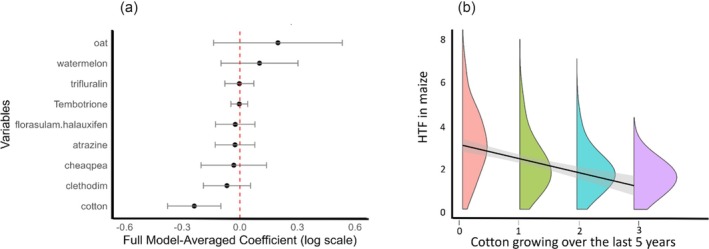
Factors associated with HTF in maize. (a) Model‐averaged coefficients with 95% confidence intervals are shown for key crop and herbicide variables, and (b) GLM predictions illustrate the relationship between cotton frequency in crop rotation and HTF in maize.

Table 4 presents the results of the GLM analysis. The model that included the historical crop frequency had the lowest AIC value, indicating the best fit. Model averaging was applied to the crop and herbicide history variables using the 95% cumulative confidence set. Figure 6(a) shows the estimated coefficients and associated variances for each crop category across the supported models. The analysis identified cotton frequency in crop history as the only variable with a significant and consistent effect on HTF in maize, supporting the hypothesis that cotton phases contribute to reduced herbicide use (H2a). Specifically, an increased frequency of cotton cultivation in the past was associated with reduced HTF in maize (estimate = −0.25, SE = 0.06, *P* < 0.001; Fig. 5(b)). Consistent with this pattern, fields without cotton in the rotation exhibited higher herbicide use intensity (mean HTF = 3.41), whereas fields with a cotton frequency of two in the rotation showed substantially lower HTF values (mean = 1.96; Supporting Information, Fig. S5). In contrast, watermelon exhibited a positive association with HTF, consistent with our hypothesis of increased herbicide use intensity. Mean HTF increased from 2.3 in fields without watermelon in the crop rotation to 3.0 in fields with a watermelon frequency of one; however, this effect was not statistically significant (Supporting Information, Fig. S6). None of the other crop categories or herbicide active ingredient variables had a significant effect on HTF. Full model selection statistics and model‐averaged coefficients, including standard errors, confidence intervals, and variable importance, are provided in Supporting Information, Table S6.

**Table 4:** Summary of the GLM analysis

Model	AIC
Historical crop frequency	464
Historical crop frequency + historical herbicide frequency	474
Historical herbicide frequency	477

### 3.3 Stage 3 – mechanistic clue finding

#### 3.3.1 Mechanism underlying the effect of cotton on HTF

**Figure 7:**
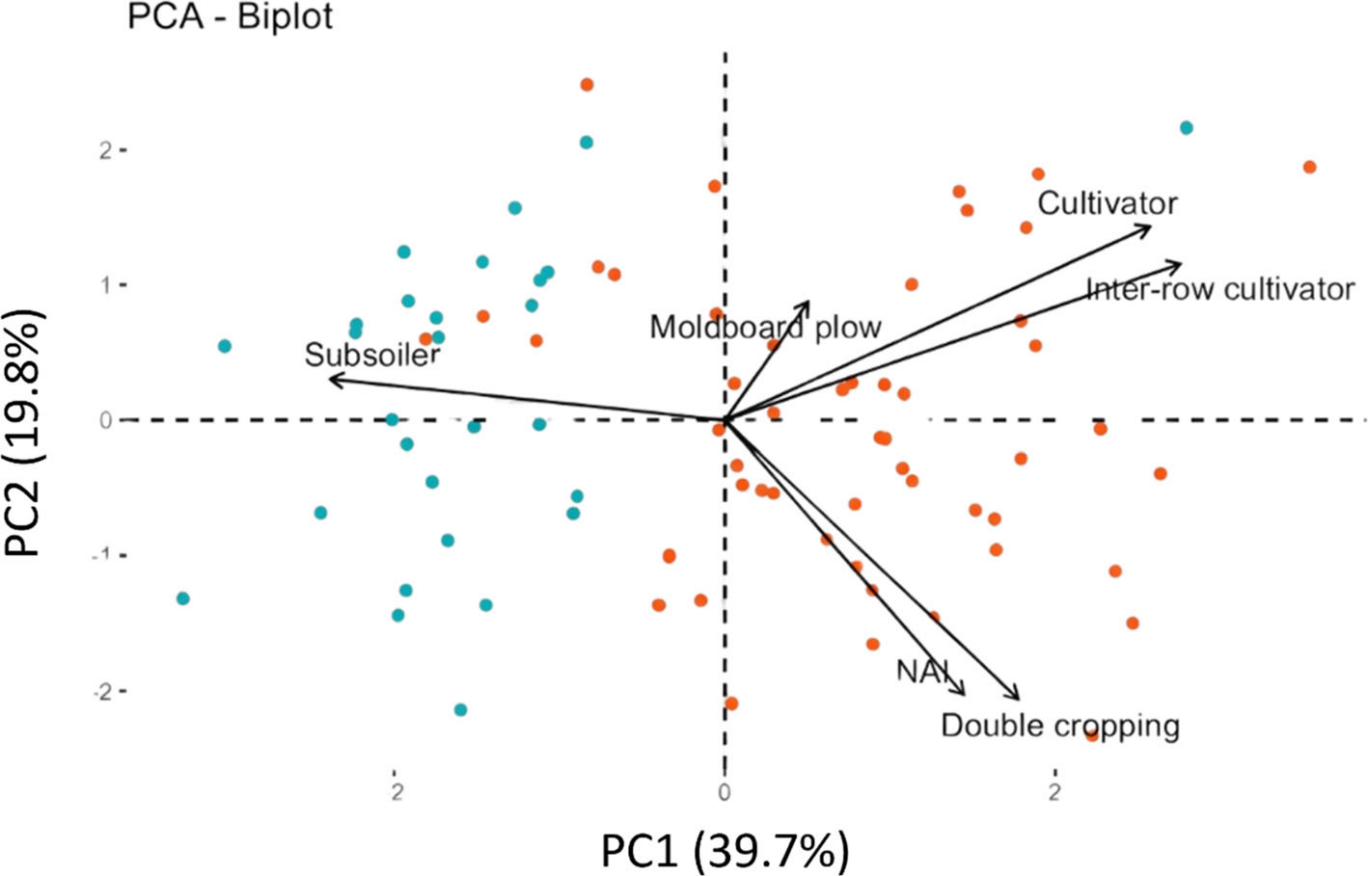
PCA biplot showing the importance of farming practices on weed suppression in maize fields, comparing fields with (orange) and without (blue) cotton in the rotation. PC1 and PC2 explain ~40% and ~ 20% of the variance, respectively.

**Figure 8:**
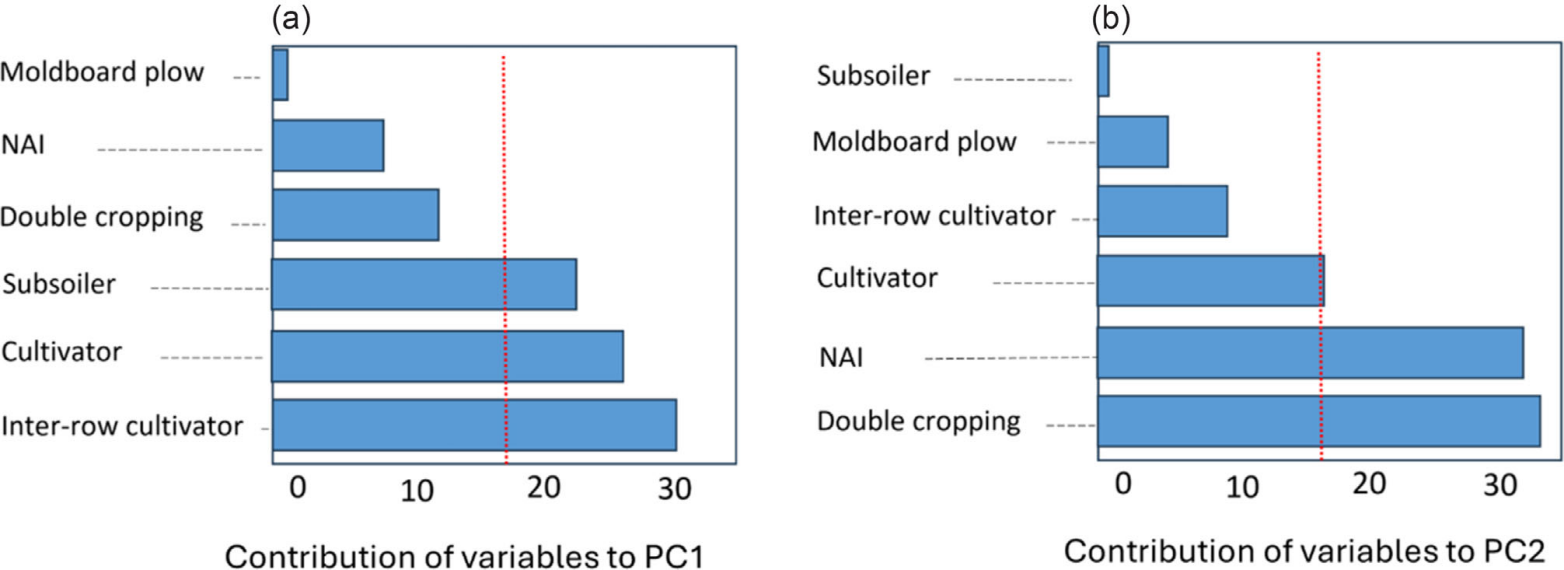
Contributions of the variables to the first two principal components, PC1 (a) and PC2 (b). Red dashed lines indicate the average contribution threshold used to identify variables with above‐average influence on each component.

PCA revealed a clear separation between maize fields with and without a history of cotton in the crop rotation (Fig. 7). The first two principal components (PC) explained 59.5% of the total variance (PC1: 39.7%, PC2: 19.8%). PC1, which primarily distinguished the two groups, one from the other, was driven by inter‐row cultivation (29.9%), cultivator use (26.0%), and subsoiler application (22.5%) (Fig. 8(a)), highlighting the role of soil tillage practices in group differentiation, in line with hypothesis H3. PC2 captured secondary variation, mainly influenced by double cropping (33.6%) and the number of active herbicide ingredients (32.6%) (Fig. 8(b)).

#### 3.3.2 Effect of cotton on HTF in maize fields over time

**Figure 9:**
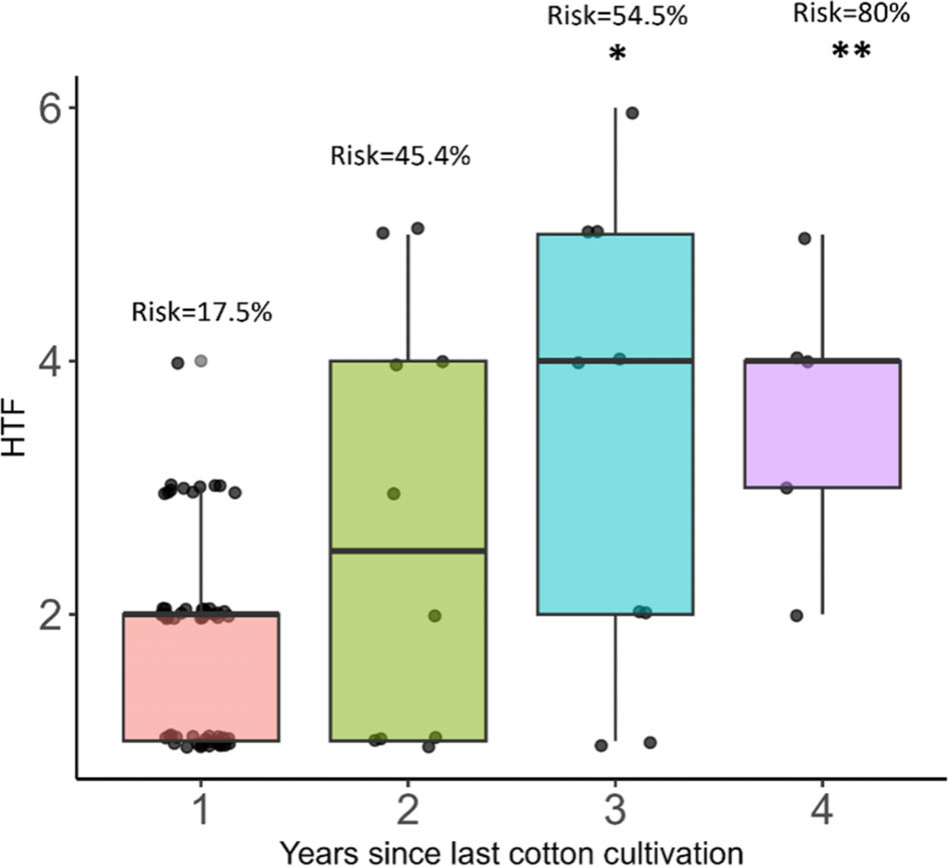
Box plot of HTF in maize fields *vs*. years since the last cotton cultivation. The risk index represents the proportion of fields with HTF > 2 relative to all fields within each time interval. Asterisks denote significance *vs*. the 1‐year reference: *P < 0.05; **P < 0.001.

HTF values in maize fields were significantly influenced by cotton being the most recent crop in the rotation (Fig. 9). No significant difference in HTF was observed between the first and second years following cotton cultivation (*P* = 0.16; Fig. 7), although the associated risk value increased 2.5‐fold (Fig. 7). In contrast, HTF values were significantly higher at 3‐ and 4‐year intervals compared to the one‐year interval (*P* = 0.006 and *P* < 0.001, respectively), with risk assessments showing a similar upward trend as the interval since cotton cultivation became larger. These findings support the hypothesis that the HTF‐reducing effect of cotton diminished within three seasons (H3b).

## 4 DISCUSSION

Results from this study demonstrate that cotton serves as a low‐infestation crop within crop rotations, potentially enhancing IWM strategies by minimizing herbicide intensity. Further, our analysis shows a significant temporal effect of the last cotton growing season on the rotation. Specifically, the risk of increased herbicide intensity was approximately threefold 3 years after the last inclusion of cotton in the rotation compared with the first following year. To the best of our knowledge, this is the first study to demonstrate the contribution of cotton, as an irrigated crop, to sustainable weed management and to quantify its effects through crop rotation under real‐world commercial farming conditions utilizing the ecoinformatics approach. This result aligns with previous studies,^41^, ^42^ which showed the importance of crop rotation to the overall weed management efficacy. However, those studies primarily focused on the overall rotation diversity and did not specifically analyze the impact of individual crops within the rotation.

### 4.1 The cotton effect

A possible mechanism through which the presence of cotton in crop rotation affects the herbicide intensity was revealed in this study, involving three cotton‐specific management components operating across the growth cycle: (i) inter‐row cultivation during early growth, (ii) deep plowing after harvest, and (iii) prolonged canopy closure.

Inter‐row cultivation is typically carried out during the early phenological stages of both the crop and the weeds, when seedlings are most vulnerable. The cutting, uprooting, or burying of emerging weeds provides effective non‐chemical control, as also demonstrated in recent field studies on early‐season mechanical tillage.^43^ According to the PCA results (Fig. 8(a)), inter‐row cultivation contributed mostly to PC1, indicating that it is the dominant factor distinguishing cotton‐inclusive from cotton‐free rotations. This conclusion aligns with the widespread use of inter‐row cultivation in Israeli cotton fields, where most growers rely on soil moisture and rainfall for germination and refrain from early‐season irrigation. The absence of drip lines early in the season allows flexible timing and inter‐row cultivation access, making it a common and cost‐effective practice, often performed twice per season to ensure crop safety (Supporting Information, Table S7). Thereby, inter‐row cultivation reduces the baseline weed density prior to chemical control^44^ and enhances weed management efficacy in subsequent crops within an IWM framework.

Deep plowing after harvest is conducted for multiple agronomic purposes, primarily to manage the pink bollworm (*Pectinophora gossypiella*), a major pest in cotton. The PCA identified deep plowing as the second most influential variable contributing to PC1 (Fig. 8(a)), highlighting its role in distinguishing cotton‐inclusive from cotton‐free rotations. This intensive tillage incorporates cotton stalks into the soil, disrupting larval development and reducing pest survival by up to 70%. In parallel, deep plowing alters the vertical distribution of weed seeds, reducing overall seedbank size by relocating seeds to deeper soil layers where emergence is less likely.^45^, ^46^ As an example, evidence from maize systems shows that tillage‐mediated weed suppression can be substantially strengthened when deep soil disturbance is combined with integrated pre‐ and post‐emergence herbicide programs, jointly reducing early establishment and late‐season weed escapes.^47^ Thus, a reduced frequency of cotton in the crop rotation, which typically involves deep plowing, can influence weed seedbank dynamics by allowing seeds to persist in the upper soil layer, increasing weed pressure and, consequently, herbicide inputs in subsequent crops.

Prolonged canopy closure – although not directly assessed in our analysis – cotton canopy closure pattern can also contribute to reduced weed pressure in cotton fields. Following crop establishment and drip irrigation installation, cotton rapidly develops a dense canopy that persists for about 100 days, roughly twice as long as that in maize.^48^, ^49^ This prolonged shading limits light and resource availability for weeds, thereby suppressing their emergence during a critical part of the season.^50^, ^51^

Beyond its contribution to IWM efficacy, the inclusion of cotton in crop rotations yields significant economic benefits. A reduction of approximately one unit in maize herbicide treatment frequency (HTF) is equivalent to eliminating one additional herbicide application. Given an average post‐emergence cost of 167 USD ha^−1^, the exclusion of cotton results in a corresponding increase in weed‐control expenditures (https://pricelist.falcha.co.il/login). These findings also raise broader considerations regarding weed management strategies beyond the cotton system. From a broader perspective, the role of soil disturbance in reducing weed infestation should be considered within sustainability trade‐offs in agricultural systems. While intensive tillage practices may reduce weed density and seedbank persistence, they can also increase soil carbon losses and CO_2_ emissions through enhanced soil disturbance and mineralization processes.^52^ In contrast, reduced tillage systems often rely more heavily on herbicides for weed control.^53^ Therefore, the balance between mechanical and chemical control is context dependent. Importantly, the weed‐suppressive effect observed in cotton‐based rotations in this study is linked to crop‐specific management practices and should be interpreted with caution when considering other cropping systems.

### 4.2 Why did herbicide intensity increase only in maize?

Herbicide intensity increased over time in maize but remained stable in cotton and watermelon (Fig. 2), partially supporting Hypothesis H1. We propose two explanations for this pattern. First, maize is strongly linked to cotton through crop rotation, whereas cotton occurred only rarely in the rotation histories of watermelon and cotton itself (Supporting Information, Table S8). Consequently, the decline in cotton cultivation between 2015 and 2021 weakened the weed‐suppressive effect of cotton in rotation, primarily affecting herbicide intensity in maize but not in cotton or watermelon (Supporting Information, Tables S9,S10). This is consistent with previous studies that have identified cotton‐maize rotations as an effective component of integrated weed and pest management.^7^, ^54^

A second explanation for the partial confirmation of H1 is the stronger reliance of maize on herbicide‐based weed control compared with cotton and watermelon.^55^, ^56^ Based on our results (Supporting Information, Table S11), maize shows low adoption of mechanical weed‐control alternatives (5%), leaving herbicides as the primary weed control strategy. In contrast, watermelon has a limited arsenal of herbicides, and its prostrate growth habit limits their use.^57^, ^58^ As a result, watermelon relies more heavily on mechanical weed control, with inter‐row cultivation adopted in approximately 30% of cases (Supporting Information, Table S11). Cotton, in turn, frequently relies on subsoiling and inter‐row cultivation as effective alternatives to herbicides, with such practices applied in about 50% of cases (Supporting Information, Table S11). Consequently, weed‐control failure in maize is more often managed through additional herbicide applications, whereas cotton and watermelon more commonly adopt weed control alternatives.

### 4.3 Why does herbicide rotation exhibit a limited association with HTF?

Herbicide rotation showed only a weak association with HTF in maize, as reflected by its limited association with historical herbicide frequency in Stage 2. This pattern was reinforced in Stage 3, where low PCA loadings for the NAI variable indicated minimal differentiation in herbicide active‐ingredient diversity between maize fields with and without cotton in the rotation. This lack of difference suggests that similar herbicide programs were applied regardless of cotton history, thereby limiting the detectable influence of herbicide rotation on HTF. This trend contrasts with previous studies reporting stronger effects of herbicide rotation on weed density and species composition.^59^, ^60^ The difference likely reflects the use of different response variables: previous studies directly measured weed abundance or community composition, whereas our analysis used herbicide intensity as a proxy for weed pressure, capturing management response rather than observed weed populations. The direct parameters are probably more ‘sensitive’ for changes in weed densities, whereas using the indirect HTF somehow blur these trends.

### 4.4 Challenges and limitations

While this study provides valuable insights into the role of cotton within crop rotations and its association with herbicide use intensity in maize fields, several challenges inherent to the ecoinformatics approach should be acknowledged.

#### 4.4.1 Data collection and coverage

Gathering field‐level records required close coordination and multiple farm visits. However, this cooperation was challenged by the COVID‐19 pandemic, which led to the cancellation of many scheduled meetings. At the outset of the study, data‐collection meetings were initiated with 20 farms to obtain historical field‐level management records. However, several farms provided limited data with insufficient historical coverage and were excluded because their historical records were incomplete or insufficient for analysis. Consequently, the final database included 11 of the 20 farms initially contacted (55% of the potential sample). Importantly, this *a priori* exclusion of farms with incomplete records meant that they were not included in any statistical analyses, ensuring that data omissions did not bias model outcomes or compromise statistical validity.

##### 4.4.1.1 The nature of inference

As with most ecoinformatics analyses, our models identify associations rather than direct causal relationships. Specifically, the study demonstrates that including cotton in crop rotations correlates with reduced HTF, but it does not directly show that cotton reduces weed occurrence. HTF served as a practical proxy for weed pressure because true weed density data or seedbank data was unavailable. In many ecoinformatics studies, the primary explanatory variables of interest are missing, and researchers must rely on indirect estimators‐often based on the management operations that farmers record most consistently.^15^, ^16^, ^61^ This limitation introduces some uncertainty into causal interpretation.

##### 4.4.1.2 Interpretation and complementarity

Despite the above‐described constraint, the observed relationships align with controlled experiments that have demonstrated the causal effects of crop and herbicide rotations, alongside the influence of soil disturbance on weed suppression. In this way, ecoinformatics and experimental studies are complementary: controlled trials isolate specific mechanisms under simplified conditions, whereas ecoinformatics confirms whether these relationships persist across broader spatial and temporal scales under real‐world management complexity.

##### 4.4.1.3 System complexity and future directions

Notably, the ‘cotton effect’ on HTF emerged despite the highly diversified Israeli cropping systems, where multiple crops are typically rotated within 5 years, conditions that could easily mask such patterns. Future work should therefore combine a large‐scale observational database with targeted field experiments to directly test whether including cotton in rotations reduces weed occurrence and, consequently, herbicide applications.

## 5 CONCLUSION

This study reveals that cotton reduces herbicide intensity when included in crop rotations. Specifically, HTF for maize fields with cotton in their rotation history was significantly lower than that for fields without cotton. The strong ‘rotational connection’ between cotton and maize suggests that changes in cotton cultivation directly affect weed management in maize. In particular, the recent increase in HTF observed in maize over the study period can be attributed to the decline in cotton cultivation, driven by global economic trends. Due to inter‐row cultivation during the initial crop stage and deep plowing after harvesting, cotton becomes a ‘lower weed infestation crop’, with the effect diminishing after 2 years. It is, therefore, important to incorporate cotton into crop rotations to maintain sustainable IWM. While the weed‐suppressive role of cotton is widely recognized in Israel, this study provides the first scientific evidence validating this hypothesis.

## CONFLICT OF INTEREST

The authors declare that they have no known competing financial interests or personal relationships that could have appeared to influence the work reported in this paper.

## AUTHOR CONTRIBUTIONS

**Shlomi Aharon**: Methodology, Data Curation, Software, Formal Analysis, Writing – Original draft preparation, Visualization. **Ran Nisim Lati**: Conceptualization, Methodology, Writing – Reviewing and Editing, Supervision, Funding acquisition. **Hanan Eizenberg**: Conceptualization, Methodology, Writing – Reviewing and Editing, Supervision, Funding acquisition. **Eitan Goldshtein**: Conceptualization, Methodology. **Yafit Cohen**: Conceptualization, Methodology, Writing – Reviewing and Editing, Supervision, Funding acquisition.

## Supporting information


**Fig. S1.** Chart showing the number of unique summer crops grown per plot over a 5‐year period.
**Fig. S2.** Frequency crops based study database.
**Fig. S3.** Boxplots of herbicide treatment frequency (HTF) in maize from 2015 to 2022.
**Fig. S4.** changes of maize (a) and cotton (b) cultivation area in Israel over 7 years from 2015 to 2022. The data used for this graph was collected from approximately 400 fields across Israel. The y‐axis shows the ratio of the total hectares for each crop in a given year to the total hectares cultivated based on the survey.
**Fig. S5.** Boxplots of herbicide treatment frequency (HTF) in maize comparing fields without cotton (0) and with a cotton frequency of two (2) in the crop rotation (over 5 years).
**Fig. S6.** Boxplots of herbicide treatment frequency (HTF) in maize comparing fields without watermelon (0) and with a watermelon frequency of 1 in the crop rotation (over 5 years).
**Table S1.** Summary of interannual rainfall characteristics (2015–2022) across three climatic regions.
**Table S2.** Summary of smooth terms from the generalized additive model using *gam. check* in maize.
**Table S3.** Generalized Additive Model (GAM) summary for cotton.
**Table S4.** Generalized Additive Model (GAM) summary for watermelon.
**Table S5.** Generalized Additive Model (GAM) summary for maize.
**Table S6.** Model‐averaged coefficients for history crop frequency predictors of HTF in maize.
**Table S7.** Mean inter‐row cultivation over season between maize and cotton.
**Table S8.** Proportion of crops growing after cotton.
**Table S9.** Model‐averaged coefficients for history crop frequency predictors of HTF in cotton.
**Table S10.** Model‐averaged coefficients for history crop frequency predictors of HTF in watermelon.
**Table S11.** Proportion of plots with repeated inter‐row cultivation.

## Data Availability

The data that support the findings of this study are available on request from the corresponding author. The data are not publicly available due to privacy or ethical restrictions.
