# Supplementary material for: Using ecoinformatics to evaluate the impact of crop and herbicide rotations on herbicide intensity
Source: Pest Manag Sci. 2026 May 23;82(9):8734–46. doi: 10.1002/ps.70925 (PMC13453158; doi:10.1002/ps.70925)
Supplement: Supplementary file 1 [file PS-82-8734-s001.docx]

**Supplementary information**

Fig. S1. Chart showing the number of unique summer crops grown per plot over a 5-year period.

Frequency (upper 90%)

Fig. S2. frequency crops based study database.

|   Figure S3. Boxplots of herbicide treatment frequency (HTF) in maize from 2015 to 2022    Fig. S4. changes of maize (a) and cotton (b) cultivation area in Israel over seven years from 2015 to 2022. The data used for this graph was collected from approximately 400 fields across Israel. The y-axis shows the ratio of the total hectares for each crop in a given year to the total hectares cultivated based on the survey.    Figure S5. Boxplots of herbicide treatment frequency (HTF) in maize comparing fields without cotton (0) and with a cotton frequency of two (2) in the crop rotation (over 5 years).    Figure S6. Boxplots of herbicide treatment frequency (HTF) in maize comparing fields without watermelon (0) and with a watermelon frequency of 1 in the crop rotation (over 5 years).  Table S1. Summary of Interannual Rainfall Characteristics (2015–2022) Across Three Climatic Regions | | | | |
| --- | --- | --- | --- | --- |
| **region** | **station** | **Mean Rainfall (mm)** | **Std. Deviation (mm)** | **Range (mm)** |
| Center | Hefer | 579 | 174 | 588 |
| North | Newe Yaar | 603 | 174 | 530 |
| North-south | Yavne | 539 | 188 | 540 |

Table S2. summary of smooth terms from the Generalized Additive Model using *gam.check* in maize.

| **Term** | **edf** | **k-index** | **p-value** |
| --- | --- | --- | --- |
| s(corn) | 1.51 | 0.95 | 0.32 |
| s(wheat) | ≈ 0 | 1.04 | 0.69 |
| s(watermelon) | ≈ 0 | 1.03 | 0.61 |
| s(cotton) | ≈ 0 | 0.89 | 0.10 |
| s(pea) | 0.67 | 0.96 | 0.36 |
| s(atrazine) | ≈ 0 | 0.92 | 0.12 |
| s(clethodim) | 0.74 | 0.95 | 0.22 |
| s(florasulam.halauxifen) | ≈ 0 | 1.04 | 0.62 |

Table S3. Generalized Additive Model (GAM) summary for cotton:

Response = HTF, Family =poisson, Link = log

| ***A. Parametric Coefficients*** | |  |  |  |
| --- | --- | --- | --- | --- |
| *Term* | *Estimate* | *Std. Error* | *t-value* | *p-value* |
| *intercept* | *1.4* | *0.34* | *4.09* | *<0.001* |
| *Farmer:haemek* | *0.19* | *0.35* | *0.57* | *0.569* |
| *Farmer:hazorea* | *-0.003* | *0.35* | *-0.009* | *0.993* |
| *Farmer:hefer* | *0.06* | *0.348* | *0.189* | *0.85* |
| *Farmer:kama* | *-0.07* | *0.348* | *0.228* | *0.820* |
| *Farmer:kefar harov* | *-0.69* | *0.423* | *-1.63* | *0.1* |
| *Farmer:megido* | *0.14* | *0.356* | *0.419* | *0.67* |
| *Farmer:saar* | *0.17* | *0.386* | *-0.466* | *0.64* |
| *Farmer:yaan* | *-0.06* | *0.353* | *-0.19* | *0.84* |
| *Farmer:yavne* | *0.12* | *0.34* | *0.35* | *0.72* |
|  |  |  |  |  |
| ***B. Smooth term*** | |  |  |  |
| *Smooth Term* | *edf* | *Ref.df* | *F* | *p-value* |
| *S (Year)* | *3.2* | *4.3* | *2.8* | *0.02* |

Table S4. Generalized Additive Model (GAM) summary for watermelon

Response = HTF, Family = poisson, Link = log

| **A. Parametric Coefficients** | |  |  |  |
| --- | --- | --- | --- | --- |
| Term | Estimate | Std. Error | t-value | p-value |
| intercept | 0.69 | 0.26 | 2.6 | 0.009 |
| Farmer:haemek | 0.26 | 0.27 | 0.96 | 0.33 |
| Farmer:hazorea | 0.44 | 0.29 | 1.47 | 0.14 |
| Farmer:hefer | 0.4 | 0.28 | 1.43 | 0.15 |
| Farmer:kama | 0.2 | 0.27 | 1.05 | 0.29 |
| Farmer:megido | 0.57 | 0.29 | 1.97 | 0.04 |
| Farmer:yaan | 0.07 | 0.29 | 0.23 | 0.81 |
| Farmer:yiron | 1.14 | 0.27 | 4.09 | <0.001 |
|  |  |  |  |  |
| **B. Smooth term** | |  |  |  |
| Smooth Term | edf | Ref.df | F | p-value |
| S (Year) | 4.8 | 6.3 | 4.9 | <0.001 |

| **A. Parametric Coefficients** | |  |  |  |
| --- | --- | --- | --- | --- |
| Term | Estimate | Std. Error | t-value | p-value |
| intercept | 1.095 | 0.14 | 7.7 | <0.001 |
| Farmer:haemek | 0.03 | 0.15 | 0.24 | 0.852 |
| Farmer:hazorea | -0.502 | 0.18 | -2.7 | 0.050 |
| Farmer:hefer | -0.67 | 0.15 | -4.3 | 0.0002 |
| Farmer:kama | -0.45 | 0.16 | -2.7 | 0.0052 |
| Farmer:kefar harov | -0.27 | 0.18 | -1.5 | 0.13 |
| Farmer:megido | -0.096 | 0.16 | -0.59 | 0.55 |
| Farmer:saar | -0.76 | 0.27 | -2.75 | 0.006 |
| Farmer:yaan | -0.35 | 0.15 | -2.23 | 0.02 |
| Farmer:yavne | -0.390 | 0.14 | -2.6 | 0.008 |
| Farmer:yiron | 0.188 | 0.15 | 1.179 | 0.23 |
| Farmer:ein harod | -0.039 | 0.21316 | -0.187 | 0.851970 |
|  |  |  |  |  |
| **B. Smooth term** | |  |  |  |
| Smooth Term | edf | Ref.df | F | p-value |
| S (Year) | 2.02 | 2.4 | 12.49 | <0.001 |

Table S5. Generalized Additive Model (GAM) summary for maize:

Response = HTF, Family = poisson, Link = log

| Table S6: Model-Averaged Coefficients for history crop frequency predictors of HTF in maize | | | | | | |
| --- | --- | --- | --- | --- | --- | --- |
| **Predictor** | **Estimate** | **Std. Error** | **Adjusted**  **SE** | **z-value** | **p-value** | **Significance** |
| Intercept | 1.238 | 0.182 | 0.182 | 6.79 | <0.001 | *** |
| Corn | –0.008 | 0.051 | 0.051 | 0.16 | 0.872 |  |
| Cotton | –0.245 | 0.070 | 0.071 | 3.46 | <0.001 | *** |
| Watermelon | 0.077 | 0.099 | 0.100 | 0.77 | 0.441 |  |
| Chickpea | –0.057 | 0.115 | 0.116 | 0.49 | 0.622 |  |
| Wheat | –0.032 | 0.058 | 0.058 | 0.55 | 0.580 |  |
| Sunflower | 0.043 | 0.093 | 0.094 | 0.45 | 0.651 |  |
| Pea | –0.040 | 0.076 | 0.076 | 0.52 | 0.604 |  |

| Table S7. Mean inter-row cultivation over season between maize and cotton \| \| \| \|; **Crop** \| **number_plots** \| **mean** \| **median** \| **sd**; **cotton** \| **303** \| **2.2** \| **2** \| **0.4**; **maize** \| **82** \| **1.2** \| **1** \| **0.45** Table S8. Proportion of crops growing after cotton | | | |
| --- | --- | --- | --- |
| **Crop** | **Previous Crop** | **Plots numbers** | **Proportion (%)** |
| **maize**  **watermelon**  **cotton** | **cotton** | **138** | **33%** |
|  |  | **42** | **9%** |
|  |  | **31** | **7%** |

| Table S9: Model-Averaged Coefficients for history crop frequency predictors of HTF in cotton | | | | | | | | | | | | |
| --- | --- | --- | --- | --- | --- | --- | --- | --- | --- | --- | --- | --- |
| **Predictor** | **Estimate** | | | **Std. Error** | | **Adjusted SE** | | **z-value** | | **p-value** | | **Significance** |
| Intercept | | 1.569 | 0.251 | | 0.252 | | 6.22 | | <0.001 | | *** | |
| Corn | | 0.032 | 0.064 | | 0.064 | | 0.50 | | 0.617 | |  | |
| Cotton | | –0.036 | 0.058 | | 0.058 | | 0.62 | | 0.532 | |  | |
| Sunflower | | 0.123 | 0.128 | | 0.128 | | 0.96 | | 0.338 | |  | |
| Watermelon | | –0.189 | 0.116 | | 0.116 | | 1.63 | | 0.103 | |  | |
| Chickpea | | 0.020 | 0.096 | | 0.097 | | 0.21 | | 0.836 | |  | |
| Pea | | –0.029 | 0.096 | | 0.096 | | 0.30 | | 0.767 | |  | |
| Wheat | | –0.022 | 0.056 | | 0.056 | | 0.39 | | 0.699 | |  | |

| Table S10: Model-Averaged Coefficients for history crop frequency predictors of HTF in watermelon | | | | | | |
| --- | --- | --- | --- | --- | --- | --- |
| Predictor | Estimate | Std. Error | Adjusted SE | z-value | p-value | Significance |
| Intercept | 1.076 | 0.212 | 0.216 | 4.99 | <0.001 | *** |
| Pea | 0.042 | 0.110 | 0.112 | 0.38 | 0.705 |  |
| Wheat | –0.058 | 0.091 | 0.092 | 0.63 | 0.527 |  |
| Watermelon | 0.002 | 0.067 | 0.068 | 0.04 | 0.972 |  |
| Chickpea | –0.036 | 0.144 | 0.146 | 0.25 | 0.806 |  |
| Corn | 0.023 | 0.064 | 0.065 | 0.35 | 0.730 |  |
| Cotton | 0.048 | 0.081 | 0.082 | 0.58 | 0.560 |  |

| Table S11. Proportion of plots with repeated inter-row cultivation | | | |
| --- | --- | --- | --- |
| **CROP** | **Plots with ≥ 1 of inter-row cultivation** | **Plots with no inter-row cultivation** | **Proportion (%)** |
| **cotton** | **115** | **107** | **51.8** |
| **watermelon** | **23** | **56** | **29.1** |
| **sunflower** | **7** | **24** | **22.6** |
| **corn** | **19** | **340** | **5.3** |
